# Research highlight: exploring the health consequences of armed conflict: the perspective of Northeast Ethiopia, 2022: a qualitative study

**DOI:** 10.1186/s12889-024-17900-8

**Published:** 2024-03-18

The Northern Ethiopian conflict has significantly impacted public health, with numerous direct and indirect results. The unrest and uncertainty have resulted in a rise in both disease and conflict-related sickness and death, impacting individuals, families, and entire communities. The conflict has also disrupted both public and private healthcare systems, leading to a sharp decrease in available health services and worsening the health crisis.

This retrospective qualitative study investigated the health consequences of the Northern Ethiopia conflict, focusing on parts of the North Wollo zone of Amhara under the control of TPLF (Tigray People’s Liberation Front) fighters for about half a year (July to December 2021). The authors collected data through detailed interviews and focus group discussions with key informants, including a total of 100 health professionals and community and religious leaders, who were directly affected by the conflict.

The conflict’s direct consequences included gunshot injuries, bomb explosions, and other forms of violence, resulting in debilitating injuries and death. Indirectly, the conflict led to a collapse in the health system, causing a lack of medical supplies, relocation of healthcare workers, disruption of food and clean water supplies, and a restriction of free movement, reducing the provision and utilization of health services. The study also emphasized the community's resilience in the face of these challenges. Patients turned to traditional medicine and home remedies or travelled to areas unaffected by the conflict to seek treatment. Health workers provided door-to-door services and used available materials like plastic as surgical gloves. Religious leaders played a crucial role in stabilizing the area, organizing health facilities, and providing emotional and material support to the community. The Northern Ethiopian conflict has left a long-lasting impact on the community’s health, with many developing psychological trauma and lifelong disabilities due to the conflict. The disruption of health improvement and preventive services, along with the destruction of health facilities, could endanger the community's health in the long run.

The findings of this study highlight the immediate need for strategies to address both the short-term and long-term impacts of the conflict. These strategies should focus on reorganizing destroyed facilities, providing rehabilitation programs for affected individuals, and re-establishing vaccination and other health improvement programs. The role of religious leaders and community cooperation in reducing the health consequences of armed conflict should also be recognized and utilized in post-conflict reconstruction and rehabilitation programs.

*The following summaries of hand-selected papers were generated by Springer Nature’s artificial intelligence tool and revised by a subject matter expert to meet Springer Nature’s standards.*

